# Diagnostic accuracy study of STANDARD TB-Feron FIA and STANDARD TB-Feron ELISA tests for tuberculosis infection diagnosis in Eastern European setting

**DOI:** 10.1016/j.jctube.2025.100518

**Published:** 2025-03-14

**Authors:** Valeriu Crudu, Dumitru Chesov, Alexandru Codreanu, Nadejda Turcanu, Nelly Ciobanu, Liuba Nepoliuc, Doina Rusu

**Affiliations:** a Chiril Draganiuc Phthisiopneumology Institute, Chisinau, Republic of Moldova; b Discipline of Pneumology and Allergology, Nicolae Testemitanu State University of Medicine and Pharmacy, Chisinau, Republic of Moldova; c Clinical Infectious Diseases, Research Center Borstel, Borstel, Germany; d German Center for Infection Research (DZIF), TTU-TB, Borstel, Germany

**Keywords:** Tuberculosis infection, IGRA, Latent tuberculosis infection

## Abstract

**Introduction:**

Tuberculosis infection (TBI) is diagnosed based on a positive immune response to *M. tuberculosis* antigens. This study aimed to evaluate both the qualitative and quantitative performance of two novel IGRA-based tests, the STANDARD E TB-Feron ELISA (TB-Feron-ELISA) and the STANDARD F TB-Feron FIA (IFN-γ) (TB-Feron-FIA), and compare their results to those of QuantiFERON-TB Gold Plus (QuantiFERON).

**Methods:**

At Chiril Draganiuc Phthisiopneumology Institute in the Republic of Moldova, we prospectively enrolled three cohorts of adults: healthy individuals with no known close contact with TB, patients with active tuberculosis (TB), and individuals with a history of TB. The active TB and past TB cohorts were used to assess the tests’ sensitivity, while the healthy group was used to evaluate specificity. Both qualitative and quantitative results from the TB-Feron ELISA and TB-Feron FIA were compared with those of QuantiFERON.

**Results:**

The TB-Feron-FIA demonstrated a sensitivity of 80.58 % (95 %CI: 71.90–87.06) in the active TB cohort and 82.93 % (95 %CI: 68.74–91.47) in the past TB cohort, with a specificity of 85.19 % (95 % CI: 73.40–92.30). The TB-Feron-ELISA showed a sensitivity of 80.58 % (95 %CI: 71.90–87.06) in the active TB cohort and 78.57 % (95 %CI: 64.06–88.29) in the past TB cohort, with a specificity of 85.19 % (95 %CI: 73.40–92.30). The agreement coefficient (κ) with QuantiFERON was 0.766 (95 %CI: 0.689–0.843) for TB-Feron-FIA and 0.809 (95 %CI: 0.739–0.880) for TB-Feron-ELISA.

**Conclusions:**

Both the TB-Feron-ELISA and TB-Feron-FIA demonstrated good diagnostic accuracy for identifying individuals with TBI, comparable to the performance of QuantiFERON.

## 1 Introduction

Tuberculosis (TB) continues to be one of the most significant public health challenges in many regions of the world, responsible for 10.6 million cases of illness and 1.4 million deaths globally in 2022 [1]. After being infected with *Mycobacterium tuberculosis* (*M. tuberculosis*), an individual may enter one of two basic scenarios: either generating an effective immune response that keeps the proliferation of *M. tuberculosis* suppressed in the body, or developing TB disease. The first of these clinical and immunological states is defined as tuberculosis infection (TBI) (formerly latent tuberculosis infection) [2]. It is estimated that one-quarter of the global population is infected with *M. tuberculosis*. Individuals with TBI may develop TB disease at any point in their life due to a weakening of the immune control over *M. tuberculosis* proliferation, which can occur as a result of various factors (e.g., HIV, immunosuppressive medications, diabetes mellitus, etc.) [3]. Therefore, identifying patients with TBI who are at high risk for developing TB disease and offering them preventive TB treatment is an important intervention for reducing TB incidence worldwide.

TBI is diagnosed based on a positive immune response to *M. tuberculosis* antigens. Currently, three main types of TBI diagnostics are endorsed by the World Health Organization (WHO). These include the classic tuberculin skin test (TST), antigen-based skin tests (TBST), and Interferon Gamma Release Assays (IGRAs). The latter are based on the in vitro quantification of Interferon-γ released by peripheral blood lymphocytes following stimulation by *M. tuberculosis* antigens such as ESAT-6 (Early Secreted Antigenic Target-6) and CFP-10 (Culture Filtrate Protein-10). Unfortunately, IGRA tests do not yet have the necessary accuracy to identify individuals with TBI who are at high risk for developing TB disease, and they have low specificity in distinguishing between TBI and TB disease [4]. To improve the diagnostic accuracy of IGRAs and facilitate their laboratory handling, a variety of alternative antigens (e.g., Rv2654, Rv3615c, and PPE family proteins) and reading tools have been proposed [5].

The STANDARD E TB-Feron ELISA (TB-Feron ELISA) and STANDARD F TB-Feron FIA (TB-Feron FIA) are two novel IGRA-based tests that use recombinant *M. tuberculosis* TB antigens (ESAT-6 and CFP-10) and apply ELISA or Fluorescent Immunoassay, respectively, for reading test results. Currently available data report on the accuracy of these tests in South American and East Asian populations; however, data on the performance of TB-Feron FIA are particularly scarce [6], [7], [8]. Both tests have demonstrated sensitivity and specificity exceeding the minimum requirements set by the WHO Target Product Profile (minimum acceptable sensitivity ≥ 75 % and specificity ≥90 %) [9].

In the present study, we aimed to assess the quantitative and qualitative results of STANDARD E TB-Feron ELISA and STANDARD F TB-Feron FIA (IFN-γ) by comparing them with the QuantiFERON-TB Gold Plus (QuantiFERON) in a population from the Republic of Moldova, an Eastern European country with a TB incidence three times higher than the European regional average (76 cases per 100,000, WHO estimate for 2023) [1].

## 2 Materials and methods

### 2.1 Study participants

For the purpose of this study, we prospectively enrolled three cohorts of adults (≥18 years old): 1) Healthy cohort – adults without clinical or imaging signs of TB, no comorbidities, and no known TB contact in the household or workplace; 2) TB cohort – patients with microbiologically confirmed pulmonary TB (via Xpert MTB/Rif Ultra and/or culture), who were treatment-naïve or had initiated TB treatment no more than two weeks prior; 3) Past TB cohort – individuals with a history of cured TB disease, in whom active TB was ruled out at enrolment based on imaging and microbiological data (i.e., negative Xpert MTB/Rif and negative *M. tuberculosis* culture). Individuals living with HIV or those receiving chronic immunosuppressive therapy were excluded from the study. HIV status was determined using rapid diagnostic tests for HIV-1 and HIV-2, following a two-step screening algorithm to ensure accurate detection. Additionally, TB patients with a negative sputum culture for *M. tuberculosis* were not included in the study.

In the absence of a gold-standard test for TB infection, these cohorts were used as surrogate reference standards to assess the sensitivity and specificity of the tests. Sensitivity was estimated in the TB and past TB cohorts, while specificity was assessed in the healthy cohort.

All study participants were consecutively enrolled at the Chiril Draganiuc Phthisiopneumology Institute (PPI) between January 1, 2023, and July 31, 2023. Patients who met the inclusion criteria for any of the study groups and provided informed consent were enrolled. PPI is a tertiary medical institution specializing in the care of patients with tuberculosis and other pulmonary diseases, located in Chisinau, Republic of Moldova.

For the study, 15 mL of peripheral blood was obtained from each participant and tested for *M. tuberculosis*-specific immune response using the STANDARD E TB-Feron ELISA (SD Biosensor, Gyeonggi-do, Republic of Korea), STANDARD F TB-Feron FIA (SD Biosensor, Gyeonggi-do, Republic of Korea), and QuantiFERON-TB Gold Plus (Qiagen, Hilden, Germany). The interpretation of the tests results was performed independently by separate investigators, each blinded to the results of the other.

### 2.2 STANDARD F TB-Feron FIA (IFN-γ)

The STANDARD F TB-Feron FIA (IFN-γ) is an in vitro diagnostic test that uses TB-specific recombinant protein antigens (ESAT-6, CFP-10, and TB 7.7) to stimulate cells in heparinized whole blood. Detection of interferon-gamma (IFN-γ) by fluorescence immunoassay (FIA) is used to identify in vitro responses to these recombinant TB antigens, which are associated with *Mycobacterium tuberculosis* infection. The TB-Feron FIA assay was performed according to the manufacturer’s instructions. Whole blood collected in lithium-heparin tubes (1 mL of whole blood per tube) was added to the three STANDARD E TB-Feron tubes: Nil, TB Ag, and the mitogen tube. The antigens evaluated were ESAT-6, CFP-10, and TB7.7. After collection, the tubes were incubated overnight at 37 °C. IFN-γ quantification was performed using the STANDARD F2400 equipment (SD Biosensor, Gyeonggi-do, Republic of Korea), an automated FIA device. The process involved sample loading into the cartridge, fluorescence intensity reading, and IFN-γ quantification, followed by interpretation. The test is performed automatically within 15 min. Results are interpreted according to the manufacturer’s instructions.

### 2.3 STANDARD E TB-Feron ELISA

The STANDARD E TB-Feron ELISA is an in vitro diagnostic test that uses TB-specific recombinant protein antigens (ESAT-6, CFP-10, and TB 7.7) to stimulate cells in heparinized whole blood. Detection of interferon-gamma (IFN-γ) by enzyme-linked immunosorbent assay (ELISA) is used to identify in vitro responses to these recombinant TB antigens, which are associated with *Mycobacterium tuberculosis* infection.

The TB-Feron assays were performed according to the manufacturer’s instructions. Whole blood collected in lithium-heparin tubes (1 mL of whole blood per tube) was added to the three tubes: the TB antigen, nil, and mitogen tubes. After overnight incubation at 37 °C, the tubes were centrifuged for 15 min at 2000 × g, and the plasma was separated. The ELISA kit was used to quantify IFN-γ in the plasma samples. The results were interpreted as positive when the IFN-γ value of the TB antigen tube minus that of the nil tube (referred to as the TB antigen-minus-nil value) was ≥0.35 IU/mL and at least 25 % of the nil value. Results were considered indeterminate if the mitogen tube value (mitogen value) minus the nil value was <0.50 IU/mL. Any other results were deemed negative.

### 2.4 QuantiFERON-TB Gold Plus

The QuantiFERON-TB Gold Plus assays were performed according to the manufacturer’s instructions. Whole blood collected in lithium-heparin tubes (1 mL of whole blood per tube) was distributed into the TB antigen tubes 1 and 2 (TB1 and TB2), the mitogen tube, and the nil tube. Both antigen tubes contained peptide antigens from the MTB-complex-associated antigens, ESAT-6 and CFP-10. After 20 h of incubation at 37 °C, the tubes were centrifuged for 15 min at 2000 × g, and the plasma was separated. An ELISA kit was used to quantify IFN-γ in the plasma samples. The results were interpreted as positive when the IFN-γ value of any TB antigen tube (TB antigen value) minus that of the nil tube (nil value) – referred to as the TB antigen-minus-nil value – was ≥0.35 IU/mL and increased by at least 25 % of the nil value. If the mitogen tube value minus the nil tube value was <0.50 IU/mL, the results were considered indeterminate. Any other results were considered negative.

### 2.5 Statistical analysis

The diagnostic accuracy of each test was evaluated by calculating sensitivity and specificity. To determine qualitative concordance between the tests, we assessed positive percent agreement, negative percent agreement, and Cohen’s κ value using a 3-by-3 crosstab analysis. Quantitative agreement between the IGRAs was evaluated using Bland-Altman plots, with mean differences and 95 % limits of agreement (average difference ± 1.96 standard deviations of the difference). A p-value of less than 0.05 was considered statistically significant. All analyses were performed using GraphPad Prism v. 9.0 (GraphPad Software, La Jolla, California, USA).

### 2.6 Ethical approval

The study was approved by the Institutional Review Board of the Chiril Dragniuc Phthisiopneumology Institute (No. 01 from 21.12.2022) and was conducted in accordance with the principles of the Declaration of Helsinki. Written informed consent was obtained from all participants.

## 3 Results

### 3.1 Subjects’ characteristics

A total of 218 participants were recruited for the study, including 57 individuals in the healthy cohort, 115 in the TB cohort, and 46 in the past TB cohort. The mean age of participants was 37.5 ± 12.4 years in the healthy cohort, 48.0 ± 13.1 years in the TB cohort, and 52.9 ± 14.9 years in the past TB cohort. All cohorts were predominantly male, with males comprising 73.7 % of the healthy cohort, 85.2 % of the TB cohort, and 73.9 % of the past TB cohort. All enrolled participants were HIV-negative.

### 3.2 Qualitative results of TB-Feron ELISA and TB-Feron FIA compared with QuantiFERON

The sensitivity of the TB-Feron FIA was 80.58 % (95 % CI: 71.90–87.06) in the TB cohort and 82.93 % (95 % CI: 68.74–91.47) in the past TB cohort. The specificity was 85.19 % (95 % CI: 73.40–92.30) (Table 1). The overall percentage agreement with QuantiFERON was 87.61 % (95 % CI: 82.49–91.68), with a Cohen's κ of 0.766 (95 % CI: 0.689–0.843). The positive percent agreement with QuantiFERON was 95.24 % (95 % CI: 90.00–97.80), while the negative percent agreement was 94.37 % (95 % CI: 86.39–97.79) (Table 2). The sensitivity of the TB-Feron ELISA was 80.58 % (95 % CI: 71.90–87.06) in the TB cohort and 78.57 % (95 % CI: 64.06–88.29) in the past TB cohort. The specificity was 85.19 % (95 % CI: 73.40–92.30) (Table 1).

**Table 1:** Diagnostic accuracy of TB-Feron FIA and TB-Feron ELISA.

	Sensitivity in active TB cohort*			**Sensitivity in pastTB cohort^#^**			**Specificity (Healthy individuals)^^^**		
	**n/N**	**%**	**95 %CI**	**n/N**	**%**	**95 %CI**	**n/N**	**%**	**95 %CI**
**TB-Feron FIA**	83/103	80.58	71.90–87.06	34/41	82.93	68.74–91.47	46/54	85.19	73.40–92.30
**TB-Feron ELISA**	83/103	80.58	71.90–87.06	33/42	78.57	64.06–88.29	46/54	85.19	73.40–92.30
**QuantiFERON**	86/113	76.11	67.47–83.03	34/44	77.27	63.01–87.16	47/56	83.93	72.19–91.31

*- patients with microbiologically confirmed pulmonary TB (via Xpert MTB/Rif Ultra and/or culture). ^#^ - individuals with a history of cured TB disease, in whom active TB was ruled out at enrollment based on imaging and microbiological data<^− adults without clinical or imaging signs of TB, no comorbidities, and no known TB contact in the household or workplace.

**Table 2:** Concordance of qualitative results of TB-Feron FIA and TB-Feron ELISA with QuantiFERON.

	**QuantiFERON**			
	**Overall agreement (%)**	**Positive agreement (%)**	**Negative agreement (%)**	**K**
**TB-Feron FIA**	87.61 (82.49–91.68)	95.24 (90.00–97.80)	94.37 (86.39–97.79)	0.766 (0.689–0.843)
**TB-Feron ELISA**	89.91 (85.12–93.57)	96.06 (91.11–98.31)	98.59 (92.44–99.93)	0.809 (0.739–0.880)

The overall percentage agreement with QuantiFERON was 89.91 % (95 % CI: 85.12–93.57), with a Cohen's κ of 0.809 (95 % CI: 0.739–0.880). The positive percent agreement with QuantiFERON was 96.06 % (95 % CI: 91.11–98.31), while the negative percent agreement was 98.59 % (95 % CI: 92.44–99.93) (Table 2).

Twenty study participants (9.2 %) had indeterminate results with TB-Feron FIA, 19 (8.7 %) with TB-Feron ELISA, and 5 (2.3 %) with QuantiFERON.

### 3.3 Concordance between quantitative results of TB-Feron FIA and TB-Feron ELISA with QuantiFERON

**Fig. 1:**
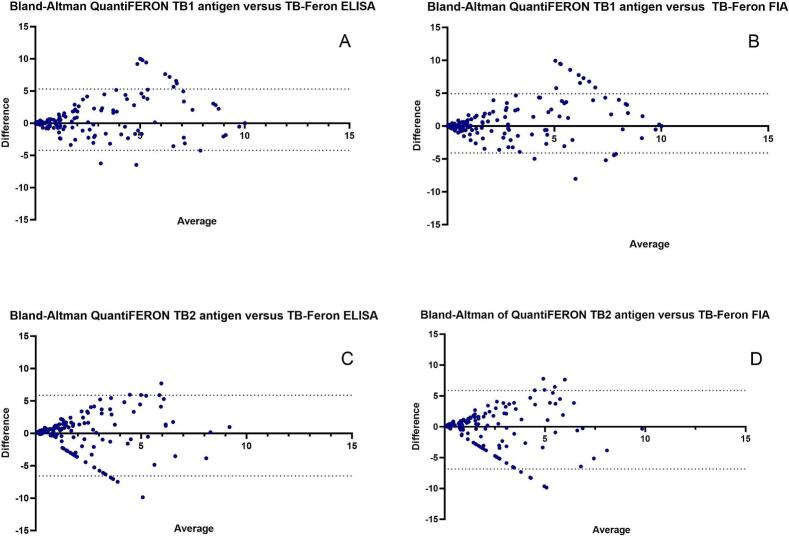
**Concordance between IGRAs results** (**Bland-Altman analysis) A)** TB Feron-ELISA compared with QuantiFERON Gold Plus TBAg1. **B)** TB Feron-FIA compared with QuantiFERON Gold Plus TBAg1. **C)** TB Feron-Elisa compared with QuantiFERON Gold Plus TBAg2. **D)** TB Feron-FIA compared with QuantiFERON Gold Plus TBAg2. Bland-Altman plots evaluate the concordance between the TB-Feron ELISA and TB-Feron ELISA FIA methods with the QuantiFERON Gold Plus assay, specifically for TBAg1 and TBAg2 antigens. In each plot, the x-axis represents the average of the two measurements being compared, while the y-axis depicts the difference between them. The solid horizontal line indicates the mean difference (bias) between the methods, and the dotted lines represent the 95 % limits of agreement (±1.96 standard deviations). The plots underscore a tight spread and close clustering of data points around the mean difference suggest a higher level of agreement between the assays.

Good concordance was observed in the quantitative results (INF-γ concentration) obtained with TB-Feron ELISA, TB-Feron FIA, and QuantiFERON. Bland-Altman analysis revealed minor mean differences in INF-γ concentrations between TB-Feron FIA and QuantiFERON. When stimulated with TB1Ag, the mean difference was 0.41 (95 % CI: −4.10 to 4.91), and when stimulated with TB2Ag, it was −0.49 (95 % CI: −6.85 to 5.87). For TB-Feron ELISA, the mean differences with QuantiFERON at TB1Ag and TB2Ag stimulation were 0.54 (95 % CI: −4.22 to 5.30) and −0.35 (95 % CI: −6.58 to 5.87), respectively. For both tests, the mean difference increased at higher INF-γ concentrations (Fig. 1).

## 4 Discussion

In the present study, we aimed to assess the diagnostic accuracy of two novel commercial versions of interferon-gamma release assays (IGRAs)—STANDARD E TB-Feron ELISA and STANDARD F TB-Feron FIA—and to compare their performance with that of QuantiFERON-TB Gold Plus. Our findings suggest that both TB-Feron ELISA and TB-Feron FIA exhibit diagnostic performance comparable to QuantiFERON-TB Gold Plus in terms of sensitivity and specificity, based on surrogate reference populations, including healthy adults without known TB contact, patients with active TB disease, and individuals with a history of cured TB. Furthermore, the quantitative results of both TB-Feron tests closely aligned with those of QuantiFERON-TB Gold Plus.

Detecting and treating tuberculosis infection (TBI) are crucial components of TB control efforts, as these measures help prevent the development of active TB [10]. Indications for tuberculosis infection testing vary based on risk factors and national guidelines. Generally, testing is recommended for individuals with known or suspected exposure to TB, such as close contacts of TB patients, immigrants from high-prevalence countries, healthcare workers, and individuals with medical conditions like HIV/AIDS or those receiving immunosuppressive treatments [11]. The choice of test—whether the tuberculin skin test (TST) or an interferon-gamma release assay (IGRA)—depends on factors such as the individual’s age, history of BCG vaccination, and resource availability [12]. IGRAs offer several advantages over the TST, including a reduced likelihood of false-positive results due to cross-reactivity with the Bacillus Calmette-Guérin (BCG) vaccine or non-tuberculous mycobacteria. Moreover, IGRAs require only a single patient visit for blood collection and result interpretation, whereas the TST requires two visits for administration and reading [5]. These advantages are also provided by the two IGRA tools assessed in this study. Previous studies have reported the non-inferiority of these tools compared to the classical QuantiFERON assay in Asian and South American populations [13], [14], [15], [16], [17], [18]. To our knowledge, this is the first study to assess the diagnostic accuracy of these two IGRA tools in an Eastern European population, a setting characterized by different population densities, tuberculosis incidence, and estimated rates of TB infection [1], [18], [19]. However, the sensitivity (78–80 %) observed for both TB-Feron assays in our study was somewhat lower than the sensitivity reported in Asian and South American settings (91–97 % for TB-Feron ELISA and 89 % for TB-Feron FIA). Similarly, the specificity (85 %) observed in our study was lower compared to previously reported rates (89–94 % for TB-Feron ELISA and 92 % for TB-Feron FIA). Nevertheless, the diagnostic performance of TB-Feron in the present study falls within the range of IGRA accuracy reported in several *meta*-analyses [20], [21], [22].

We also observed a higher rate of indeterminate results for both TB-Feron ELISA (8.7 %) and TB-Feron FIA (9.2 %) compared to QuantiFERON (2.3 %). On average, indeterminate IGRA results are reported in 1 % to 10 % of cases, with higher rates often seen in individuals with HIV or other immunosuppressive conditions [23]. In immunocompetent populations, indeterminate rates typically range from 1 % to 5 %, consistent with the rate observed for QuantiFERON in our study [24]. Since all tests in the present study were performed on the same individuals and samples and handled by the same personnel, it is reasonable to attribute the higher indeterminate rate for TB-Feron tests to specific technical features of these assays. This suggests the need for further refinement of these tools in future iterations.

Both TB-Feron FIA and TB-Feron ELISA offer potential advantages over the TST, which remains widely used in Eastern European countries. Limited use of IGRA in resource limited setting a determined by high implementation costs for laboratory infrastructure, including incubators and ELISA readers. As well timely blood sample processing (often within 8–16 h) is also difficult in areas with limited transport and laboratory capacity. Additionally, the high cost of test kits and equipment maintenance restricts the widespread adoption of these tests in such environments. Addressing these logistical and financial barriers is essential for the broader implementation of IGRAs in resource-limited settings.

An eventual consideration for implementation of TB-Feron FIA should also take into account the potential benefit of a shorter turnaround time, which allows for quicker diagnosis and may provide further advantages for TB-Feron FIA over ELISA-based IGRAs [25]. Although our study did not specifically assess this aspect, the assumption is supported by the simplified fluorescence-based reading method of TB-Feron FIA, which requires minimal manual handling compared to the more labor-intensive ELISA procedure.

While IGRAs reduce the risk of false-positive results associated with BCG vaccination, an important aspect in Eastern Europe, where BCG vaccination is administered at birth to more than 90 % of newborns, the potential for false negatives remains a concern, particularly in immunocompromised individuals or those with a low bacterial burden [26]. This issue is especially relevant given the increasing rates of TB/HIV co-infection in the region (e.g., in the Republic of Moldova, the TB/HIV co-infection rate has increased over the past decade, rising from 7 % in 2015 to 13 % in 2025) [1].

Despite the technical limitations of IGRAs, such as the need for laboratory infrastructure and trained personnel, an additional important limitation of these tests is given by the fact that they cannot differentiate between tuberculosis infection and active TB. Moreover, IGRAs are unable to predict the progression of tuberculosis infection to active disease [27], [28]. These limitations apply to both tests assessed in the present study. Therefore, there is a need for novel diagnostic tools based on alternative biomarkers capable of distinguishing between tuberculosis infection and active TB, as well as predicting disease progression. For example, identifying and measuring specific proteins or metabolites produced by *Mycobacterium tuberculosis* or the host immune response may facilitate the development of more accurate diagnostic tests. Advancements in imaging techniques, such as PET scans or next-generation radiography, could also provide valuable tools for differentiating between active TB and tuberculosis infection [5].

The data from the present study should be interpreted with several limitations in mind. Notably, the study did not include children or individuals living with HIV, both of whom are explicitly targeted for TB preventive therapy in current guidelines [29]. These populations are at higher risk of TB infection and often experience higher rates of false-negative results, higher indeterminate rates, and technical challenges in testing. Another limitation of our study is the predominance of male participants, which may limit the generalizability of the findings to female populations. These trends underscore the need for further studies investigating the performance of TB-Feron tests in these populations, as well as cost-effectiveness analyses to support feasibility of the implementation of these diagnostic technologies in clinical practice.

## 5 Conclusion

Used in an East European population both TB-Feron ELISA and TB-Feron FIA had a good diagnostic accuracy for tuberculosis infection comparable with that of other classical IGRAs.

## CRediT authorship contribution statement

**Valeriu Crudu:** Writing – review & editing, Writing – original draft, Methodology, Conceptualization. **Dumitru Chesov:** Writing – original draft, Methodology, Formal analysis, Conceptualization. **Alexandru Codreanu:** Investigation. **Nadejda Turcanu:** Investigation. **Nelly Ciobanu:** Investigation. **Liuba Nepoliuc:** Writing – review & editing, Investigation. **Doina Rusu:** Writing – review & editing.

## Funding

Diagnostic devices and reagent kits was provided free of charge by SD Biosensor. The funder did not have any additional role in data collection and analysis, decision to publish, or preparation of the manuscript.

## Declaration of competing interest

The authors declare that they have no known competing financial interests or personal relationships that could have appeared to influence the work reported in this paper.

## References

[1] World Health Organization. Global tuberculosis report 2023. Glob Tuberc Rep. 2023.

[2] Hunter R.L. (2016). Tuberculosis as a three-act play: A new paradigm for the pathogenesis of pulmonary tuberculosis. Tuberculosis.

[3] Lange C., Dheda K., Chesov D., Mandalakas A.M., Udwadia Z., Horsburgh C.R. (2019). Management of drug-resistant tuberculosis. Lancet.

[4] Lange C., Mandalakas A.M., Kalsdorf B., Denkinger C.M., Sester M. (2016). Clinical application of interferon-γ release assays for the prevention of tuberculosis in countries with low incidence. Pathog Immun.

[5] Goletti D., Delogu G., Matteelli A., Migliori G.B. (2022). The role of IGRA in the diagnosis of tuberculosis infection, differentiating from active tuberculosis, and decision making for initiating treatment or preventive therapy of tuberculosis infection. Int J Infect Dis.

[6] Lee D.G., Kang J., Jung J., Kim T., Kim J., Lee H. (2023). Comparison of the Standard E TB-Feron ELISA and QuantiFERON-TB Gold PLUS assays: the advantageous use of whole recombinant protein antigens for latent tuberculosis diagnosis. Lett Appl Microbiol.

[7] Saint-Pierre G, Conei D, Cantillana P, Raijmakers M, Vera A, Gutiérrez D, et al. Comparison of two tuberculosis infection tests in a South American Tertiary Hospital: STANDARD F TB-Feron FIA vs. QIAreachTM QuantiFERON-TB. Diagnostics (Basel, Switzerland). 2023;13(6). 10.3390/diagnostics13061162 PMC10046924 36980470

[8] Yoo I.Y., Lee J., Choi A.R., Jun Y.H., Park Y.J., Lee H.Y. (2021). Comparative evaluation of standard E TB-Feron ELISA and QuantiFERON-TB Gold Plus assays in patients with tuberculosis and healthcare workers. Diagnostics (Basel, Switzerland).

[9] World Health Organization. Consensus meeting report: development of a Target Product Profile (TPP) and a framework for evaluation for a test for predicting progression from tuberculosis infection to active disease. WHO Publ. 2017;22.

[10] World Health Organisation. The end TB Strategy global strategy and targets for tuberculosis prevention, care and control after 2015 a. Geneva; 2014.

[11] Organizat WH. WHO consolidated guidelines on tuberculosis. Module 1: Prevention. Tuberculosis preventive treatment. Tuberculosis, Lung Diseases, HIV Infection. 2021. p. 86–92.

[12] Chitnis A.S., Jaganath D., Gish R.G., Wong R.J. (2021). Diagnosis and treatment of latent tuberculosis infection. Am J Gastroenterol.

[13] Benachinmardi K., Sampath S., Rao M. (2021). Evaluation of a new interferon gamma release assay, in comparison to tuberculin skin tests and quantiferon tuberculosis goldplus for the detection of latent tuberculosis infection in children from a high tuberculosis burden setting. Int J Mycobacteriol.

[14] Jung J., Jhun B.W., Jeong M., Yoon S.J., Huh H.J., Jung C.W. (2021). Is the new interferon-gamma releasing assay beneficial for the diagnosis of latent and active mycobacterium tuberculosis infections in tertiary care setting?. J Clin Med.

[15] Lee D.G., Kang J., Jung J., Kim T., Kim J., Lee H. (2023). Comparison of the Standard E TB-Feron ELISA and QuantiFERON-TB Gold PLUS assays: the advantageous use of whole recombinant protein antigens for latent tuberculosis diagnosis. Lett Appl Microbiol.

[16] Yoo I.Y., Lee J., Choi A.R., Jun Y.H., Lee H.Y., Kang J.Y. (2021). Comparative evaluation of standard E TB-Feron ELISA and QuantiFERON-TB gold plus assays in patients with tuberculosis and healthcare workers. Diagnostics (Basel, Switzerland).

[17] Kweon O.J., Lim Y.K., Kim H.R., Kim T.H., Lee M.K. (2019). Evaluation of standard e Tb-Feron enzyme-linked immunosorbent assay for diagnosis of latent tuberculosis infection in health care workers. J Clin Microbiol.

[18] Uddin M., Islam A., Jabin M., Alam T., Khair S., Ferdous J. (2024 Sep). Comparative evaluation of diagnostic performance: standard E TB Feron ELISA vs QuantiFERON-TB gold plus for latent tuberculosis infection detection in diverse risk groups in Bangladesh. Infect Drug Resist.

[19] Cohen A., Mathiasen V.D., Schön T., Wejse C. (2019). The global prevalence of latent tuberculosis: a systematic review and meta-analysis. Eur Respir J.

[20] Wen A., Leng E.L., Liu S.M., Zhou Y.L., Cao W.F., Yao D.Y. (2022). Diagnostic accuracy of interferon-gamma release assays for tuberculous meningitis: a systematic review and meta-analysis. Front Cell Infect Microbiol.

[21] Petnak T., Eksombatchai D., Chesdachai S., Lertjitbanjong P., Taweesedt P., Pornchai A. (2022). Diagnostic accuracy of interferon-gamma release assays for diagnosis of smear-negative pulmonary tuberculosis: a systematic review and meta-analysis. BMC Pulm Med.

[22] Peng L., Ma W., Zhong L., Yang J., Wu H., Zhu L. (2024). Diagnostic accuracy of Mycobacterium tuberculosis antigen-based skin tests (TBSTs) for tuberculosis infection compared with TST and IGRA: a network meta-analysis. Pathogens.

[23] Zhou G., Luo Q., Luo S., Chen H., Cai S., Guo X. (2023). Indeterminate results of interferon gamma release assays in the screening of latent tuberculosis infection: a systematic review and meta-analysis. Front Immunol.

[24] Oni T., Gideon H.P., Bangani N., Tsekela R., Seldon R., Wood K. (2012). Risk factors associated with indeterminate gamma interferon responses in the assessment of latent tuberculosis infection in a high-incidence environment. Clin Vaccine Immunol.

[25] Saint-Pierre G., Conei D., Cantillana P., Raijmakers M., Vera A., Gutiérrez D. (2023). Comparison of two tuberculosis infection tests in a South American Tertiary hospital: STANDARD F TB-Feron FIA vs. QIAreachTM QuantiFERON-TB. Diagnostics.

[26] Yamasue M., Komiya K., Usagawa Y., Umeki K., Nureki S.i., Ando M. (2020). Factors associated with false negative interferon-γ release assay results in patients with tuberculosis: a systematic review with meta-analysis. Sci Reports.

[27] Chesov D., Lange C., Daduna F., Crudu V., Preyer R., Ernst M. (2014). Additional analysis of antigen-specific Interleukin-2 release does not improve the performance of IGRAs for the differentiation of different states of mycobacterium tuberculosis infection. Eur Respir J.

[28] Goletti D., Sanduzzi A., Delogu G. (2014). Performance of the tuberculin skin test and interferon-γ release assays: an update on the accuracy, cutoff stratification, and new potential immune-based approaches. J Rheumatol Suppl.

[29] Christof C, Nußbaumer-Streit B, Gartlehner G. WHO Guidelines on Tuberculosis Infection Prevention and Control. Vol. 82, Gesundheitswesen. 2020. p. 885–9. 10.1055/a-1241-4321 32977345

